# Job satisfaction of general practitioners: a cross-sectional survey in 34 countries

**DOI:** 10.1186/s12960-021-00604-0

**Published:** 2021-04-27

**Authors:** Emiel J. Stobbe, Peter P. Groenewegen, Willemijn Schäfer

**Affiliations:** 1 Nivel – Netherlands Institute for Health Services Research, PO Box 1568, 3500 BN Utrecht, The Netherlands; 2 Present Address: Trimbos Institute, Da Costakade 45, 3521VS Utrecht, The Netherlands; 3 Department of Sociology, Department of Human Geography, Utrecht University, Utrecht, The Netherlands; 4 Department of Surgery, Northwestern University, Feinberg School of Medicine, 633 N. St Clair Street, Chicago, IL 60611 USA

**Keywords:** Job satisfaction, General practice, International comparison, Social Production Function Theory

## Abstract

**Background:**

Job satisfaction of general practitioners (GPs) is important because of the consequences of low satisfaction for GPs, their patients and the health system, such as higher turnover, health problems for the physicians themselves, less satisfied patients, poor clinical outcomes and suboptimal health care delivery. In this study, we aim to explain differences in the job satisfaction of GPs within and between countries.

**Methods:**

We performed a secondary analysis of cross-sectional survey data, collected between 2010 and 2012 on 7379 GPs in 34 (mostly European) countries, as well as data on country and health system characteristics from public databases. Job satisfaction is measured through a composite score of six items about self-reported job experience. Operationalisation of the theoretical constructs includes variables, such as the range of services GPs provide, working hours, employment status, and feedback from colleagues. Data were analysed using linear multilevel regression analysis, with countries and GPs as levels. We developed hypotheses on the basis of the Social Production Function Theory, assuming that GPs ‘produce’ job satisfaction through stimulating work that provides a certain level of comfort, adds to their social status and provides behavioural confirmation.

**Results:**

Job satisfaction varies between GPs and countries, with high satisfaction in Denmark and Canada (on average 2.97 and 2.77 on a scale from 1–4, respectively) and low job satisfaction in Spain (mean 2.15) and Hungary (mean 2.17). One-third of the total variance is situated on the country level, indicating large differences between countries, and countries with a higher GDP per capita have more satisfied GPs. Health system characteristics are not related to GP job satisfaction. At the GP and practice level, performing technical procedures and providing preventive care, feedback from colleagues, and patient satisfaction are positively related to GP job satisfaction and working more hours is negatively related GP job satisfaction.

**Conclusion:**

Overall and in terms of our theoretical approach, we found that GPs are able to ‘produce’ work-related well-being through activities and resources related to stimulation, comfort and behavioural confirmation, but not to status.

**Supplementary Information:**

The online version contains supplementary material available at 10.1186/s12960-021-00604-0.

## Background

There is widespread dissatisfaction among general practitioners (GPs) in several countries and the share of GPs in the physician workforce is decreasing, despite the growing importance of primary care [1]. It is important that health care professionals enjoy their work. It not only affects themselves, but also their patients and health care delivery as a whole. Research on GPs as well as physicians in general, shows that those with higher job satisfaction have a lower likelihood of burnout [2], stay longer in their job [3] and experience less job stress, are more involved in decisions that affect work and perceive their work as interesting [4]. On the other hand, low job satisfaction can have some serious negative consequences, such as less satisfied patients and higher turnover [5], poor clinical outcomes and suboptimal health care delivery [6], and health problems for the physicians themselves [7].

In this article, we aim to explain variation in job satisfaction of GPs between countries and GPs/GP practices. We performed a secondary analysis of a large dataset that includes a measure of GP job satisfaction in 34 (mainly European) countries, collected in the ‘Quality and Costs of Primary Care in Europe’ (QUALICOPC) study [8]. This adds an international perspective to the existing literature. Personal and practice characteristics of GPs only explain a small portion of the overall variation in job satisfaction [9]. This suggests that part of the explanation for variation in job satisfaction is to be found at the level of countries and their health systems. Previous studies that have shown differences in job satisfaction between GPs from different countries, have mostly involved only two or three countries or had only small samples [10–12]. By using a multilevel approach and including 34 countries, measuring both country-level and GP-level influences on job satisfaction, this study adds by providing a more comprehensive international perspective. Knowing how much of the variation in GP job satisfaction is between countries and what it is in countries and their health system that explains this variation, may provide a starting point for policy development.

We will answer the following research questions:

- How satisfied are GPs with their job in 34 countries?
- How can we explain differences in the job satisfaction of GPs within and between countries?

We will develop hypotheses to explain variation in job satisfaction on the basis of Social Production Function (SPF) theory [13, 14]. To test the hypotheses, we performed a secondary analysis on data from the international QUALICOPC study [15]. In this study, job satisfaction is measured using a scale of six statements about satisfaction with different aspects of GPs work. These statements are about whether they think their work is useful, whether it is interesting, whether they feel like their administrative tasks take up too much time, whether their work causes too much stress, whether they regard GPs to be respected, and whether there is a good balance between their work and the rewards they receive for it.

### Theoretical framework and hypotheses

SPF theory is a theory of goal-oriented behaviour, which assumes that people try to optimise the achievement of universal goals, given their resources and constraints, in order to produce their own well-being [13, 14]. In line with this theory, we define GP job satisfaction as ‘the production of positive feelings regarding aspects of their work as GP, influenced by the resources that are available and the present circumstances’. Work is related to the production of well-being because it brings people a certain level of stimulation, social relationships, and a sense of belonging and meaning [16].

The SPF theory assumes that people act goal-oriented against the background of their ideas and knowledge about the appropriate means and ends to achieve this [17]. It depends on the social and institutional context in what ways, i.e. through which instrumental goals, they can reach the universal goals of physical and social well-being. Physical well-being is acquired through the right mix of two instrumental goals: stimulation and comfort. Social well-being is acquired through three instrumental goals: status, behavioural confirmation and affection [18].

SPF theory has been applied previously to workload and decisions about the allocation of time of GPs. In this application, it is assumed that GPs in their professional work strive after both personal goals and benefits for their patients, and it is suggested that the realisation of these goals increases their job satisfaction and self-esteem [19]. Examples of important instruments for GPs to produce physical well-being are income and leisure time, while an important instrumental goal for social approval is whether they provide quality care for their patients. Affection is not considered as an important instrumental goal for the production of satisfaction in the context of work, since it seems more likely that affection is acquired through relationships with family members and loved ones rather than through working relationships. Table 1 shows how the hierarchy of social production functions can be applied to ways to produce GP job satisfaction. In the sections that follow, we will develop hypotheses about the way GPs can reach the four instrumental goals in their work. We should note that it is not always possible to subsume a hypothesis under one and only one instrumental goal. We also note that—given we did a secondary analysis—we only formulated hypotheses about activities or resources about which the QUALICOPC study contains information.

**Table 1:** Application of SPF theory to GP job satisfaction

	GP job satisfaction				
Universal goals	Work-related physical well-being		Work-related social well-being		
Instrumental goals	Stimulation	Comfort	Status	Behavioural confirmation	Affection
Examples of ways to realise the instrumental goals	Varied and challenging work	Comfortable working conditions	Autonomy	Relationships with colleagues and patients	[not used in this study]

#### Stimulation

GPs can realise stimulating work, and thereby produce job satisfaction, by providing a wide variety of different services.

H1. GP job satisfaction is higher for GPs who provide a broader range of services.

The provision of a broad service profile is (among others) constrained by the resources in terms of equipment GPs have at their disposal [20]. A lack of equipment can diminish the range of services GPs can provide to their patients [21].

H2. Job satisfaction is higher for GPs who have more medical equipment at their disposal.

Another way for GPs to have more challenging and varied work is to have other paid activities next to working as a GP. GPs who participate in teaching were more satisfied with their work than GPs who did not [22]. Involvement in other professional activities can serve multiple instrumental goals at the same time: more stimulation from a wider variety of tasks; more status from improving their skills; and more behavioural confirmation from feedback and support from, e.g. students.

H3. GP job satisfaction is higher for GPs who have other paid professional activities.

The practice location may be related to GP job satisfaction for two reasons. Firstly, GPs in rural areas have a more varied practice because there are less alternatives to their patients, such as hospitals [23, 24]. Secondly, people in rural areas are more attached to and socially integrated in the community than people in urban areas [25]. Thus, GPs in rural areas may be more satisfied due to more opportunities for both stimulation and behavioural confirmation.

H4. GP job satisfaction is higher for GPs who work in rural areas.

#### Comfort

We expect that GPs experience less comfort in their job when they work more hours. Several studies have shown that long working hours and working full-time are strongly associated with lower job satisfaction for GPs [4, 23, 26–28].

H5. GP job satisfaction is higher for GPs who work less hours.

GPs report low rates of satisfaction with administrative tasks [29]. Moreover, administrative work is one of the aspects that is least appreciated by GPs when compared to other physicians [30].

H6. GP job satisfaction is higher for GPs who spend less time on administrative work.

Although most GPs acknowledge that out-of-hours work is an important aspect of their profession, they generally consider it as very demanding [31] and especially night shifts are a major source of stress [32].

H7. GP job satisfaction is higher for GPs who spend less time on out-of-hours work.

The use of information technology has the potential to reduce the workload of GPs and may provide assurance when making decisions [33].

H8. GP job satisfaction is higher for GPs who use a computer for more different purposes in their practice.

Workers have lower levels of job strain during and a few weeks after their vacation [34, 35]. This seems to apply to physicians as well; physicians who have more than two vacations per year are more satisfied with their work [36].

H9. GP job satisfaction is higher for GPs who take more vacation.

Good physical working conditions, such as the cleanliness of the workplace, adequate tools and equipment and pleasant lighting, make it easier for people to do their jobs comfortably and efficiently [37]. Unfavourable working conditions can have a negative impact on the mental and physical well-being of workers, negatively affecting their job satisfaction [38]. We assume that this applies to GP as well.

H10. GP job satisfaction is higher for GPs whose practice provides a more pleasant physical work environment.

How out-of-hours care at health system level is organised, may affect GP job satisfaction. The introduction of GP cooperatives in a number of countries led to a reduction of their workload and increased their job satisfaction [27, 39, 40]. Organisation is often preferred through a GP cooperative that combines size of scale advantages with organisational features of strong primary care, such as good accessibility, continuity and coordination of care [41]. We expect GPs to be more satisfied with GP-based models than with hospital- and national-based models, and that large doctor based models are more satisfactory than small GP-based models in terms of workload.

H11. GP job satisfaction is lowest in countries where hospital- and national-based models are dominant, higher in countries where GP-based models are dominant, and highest in countries where large GP-based models are dominant.

#### Status

Self-employed people have a greater likelihood of being satisfied with their job than salaried employees [42]. This has been found for self-employed GPs, as opposed to salaried GPs, as well [43]. The independence that comes with self-employment gives autonomy and encourages GPs to provide a broader range of services [44].

H12. GP job satisfaction is higher for GPs who are self-employed than for GPs who are salaried.

Income of GPs differs between countries and reflects their relative status, the importance that is attached to the services of GPs, and their bargaining power [45].

H13**.** GP job satisfaction is higher in countries where GP income is equal or higher compared to other medical specialists.

In most European countries, GPs are the most important providers of primary care [8]. The stronger a country’s primary care system is, the more important the role of GPs within the system is [46] and the higher their status will be.

H14. GP job satisfaction is higher in countries with stronger primary care.

#### Behavioural confirmation

Feedback provides recurrent evaluations of work performance and it gives workers a better sense of what is expected of them and helps them to develop the necessary skills or judgements for the job [47]. Having good relationships with colleagues increases GP job satisfaction [48–50].

H15. GP job satisfaction is higher for GPs who receive feedback from their colleagues.

Patients can be a source of behavioural confirmation when they show their appreciation for the care their GP has provided. There is a positive relationship between patient satisfaction and GP job satisfaction [51, 52]. Furthermore, some of the main sources of lower satisfaction for GPs are increased demands by patients [53] and patients’ expectations [54].

*H16*. *GP job satisfaction is higher for GPs who have more satisfied patients*.

The mode of practice, solo or group, may be related to job satisfaction, although the direction is indeterminate. A shared practice with multiple GPs provides opportunities for behavioural confirmation [55]. However, GPs in solo practices have more autonomy and less bureaucratic controls than GPs in shared practices.

H17**.** GP job satisfaction is related to the mode of practice.

GPs in countries where patients are listed with a specific GP or practice, have more opportunities to receive behavioural confirmation from their patients. The list system allows GPs to get to know their patients and the patients’ families better, resulting in better continuity of care [56]. In such systems patients are more likely to be familiar with a particular GP which is associated with higher patient satisfaction with care [57].

H18**.** GP job satisfaction is higher is countries with a patient list system.

## Methods

### Data

The data for this study were collected in the QUALICOPC study between 2010 and 2012 [8], for which four questionnaires were developed: one for GPs, one for patients about their experiences with their GP, another for patients about their values regarding primary care, and finally one about the practice. The patient questionnaires were administrated by field workers. The questions were derived from existing, validated questionnaires [44, 58, 59]. Development of the questionnaires consisted of four phases: a search for existing questionnaires, the classification and selection of relevant questions, shortening of the questionnaires and a pilot survey. Furthermore, data about primary health care were gathered at the national level in available databases [60]. The main source of data for this secondary analysis was the GP questionnaire. Patient satisfaction was derived from the Patient Experiences questionnaire, and the condition of the practice premises from the Fieldworker questionnaire.

The QUALICOPC study was conducted in 34 (mostly European) countries. The target response was 220 GPs per country, except for the four smallest countries where the target was 75. The aim was to draw a nationally representative sample of GPs with one GP per practice in each country. The information on the actual implementation of the study was provided by the national coordinators. The participation rate was calculated based on their information on the numbers of GPs invited and the actual number in the database. The estimated participation rates vary from less than 10% in Austria, Belgium, Germany, Ireland and Sweden to over 70% in Cyprus, Greece, Iceland, Malta and Spain, with an average of 30% [61]. The GP questionnaire was filled in by 7,414 GPs and the Patients Experiences questionnaire was filled in by 63,887 patients. Further details about the development of the questionnaires and the data collection have been published elsewhere [60, 61]. Ethical approval for the QUALICOPC study was acquired in accordance with the legal requirements in each country [62].

### Measurements

The question that was used to measure the dependent variable, job satisfaction, was derived from the European Task Profile Study [20], an international study on the variation in the tasks of GPs in Europe. In this question GPs were asked to what extent they agree with six statements and answering categories ranging from 1 (strongly agree) to 4 (strongly disagree). The statements are:

- I feel that some parts of my work do not really make sense
- My work still interests me as much as it ever did
- My work is overloaded with unnecessary administrative detail
- I have too much stress in my current job
- Being a GP is a well-respected job
- In my work there is a good balance between effort and reward.

The items were (re)coded in way that higher satisfaction is indicated by a higher score. Each GP received a score on a scale of 1 (low job satisfaction) to 4 (high job satisfaction).

GP- and practice-level independent variables were operationalised using data from the QUALICOPC GP questionnaire, except for ‘patient satisfaction’ and ‘physical work environment’, for which data from, respectively, the patient experience questionnaire and the fieldworker questionnaire were used. Country-level variables were operationalised with data from external sources to the QUALICOPC study [41, 63, 64]. The operationalisations of the independent variables are shown in the Additional file 1: Tables S1–S3 (for the GP and practice variables) and 2 (for the country variables).

### Statistical analysis

We used multilevel random effects linear regression analysis to take the nested structure of the data into account [65]. In the main analysis we distinguished between GPs or practices nested within countries. Moreover, the dependent variable job satisfaction was constructed through an ecometric analysis, with items at the lowest level, nested within GPs and countries [65]. Since only one GP per practice was invited to participate, GPs and practices are at the same level. To prevent attenuation of the number of observations due to missing cases, we used dummy variables for missing values (not reported in tables).

The following modelling strategy was used. We first estimated an empty model (Model 0) with the variation in job satisfaction at GP and country level. Subsequently, GP/practice characteristics were added in four steps, according to the instrumental goals in SPF theory: stimulation, comfort, status and behavioural confirmation. In the model with the behavioural confirmation variables, we also included the interaction between patient satisfaction and shared practice. After adding the corresponding groups of variables to the model one group at a time, they are all added simultaneously. This results in Model 5, which is used as a baseline model for the evaluation of the country-level variables. Each country model contains no more than three variables on a country level, since the analysis only contains 34 countries. This means that we tested the country-level variables in separate models (Model 6–10), with GDP per capita as a control variable.

We will report Model 0 and Model 5, since Models 1–4 do not add much information compared to Model 5. In reporting the country variable coefficients we do not report the GP/practice-level coefficients, because they are hardly affected by including the country-level variables. A significance level of *p* < 0.05 for GP/practice variables and *p* < 0.10 for country-level variables (because of the small number of observations at country level) was used.

The data were analysed with Stata 15 (command: mixed).

## Results

**Fig. 1:**
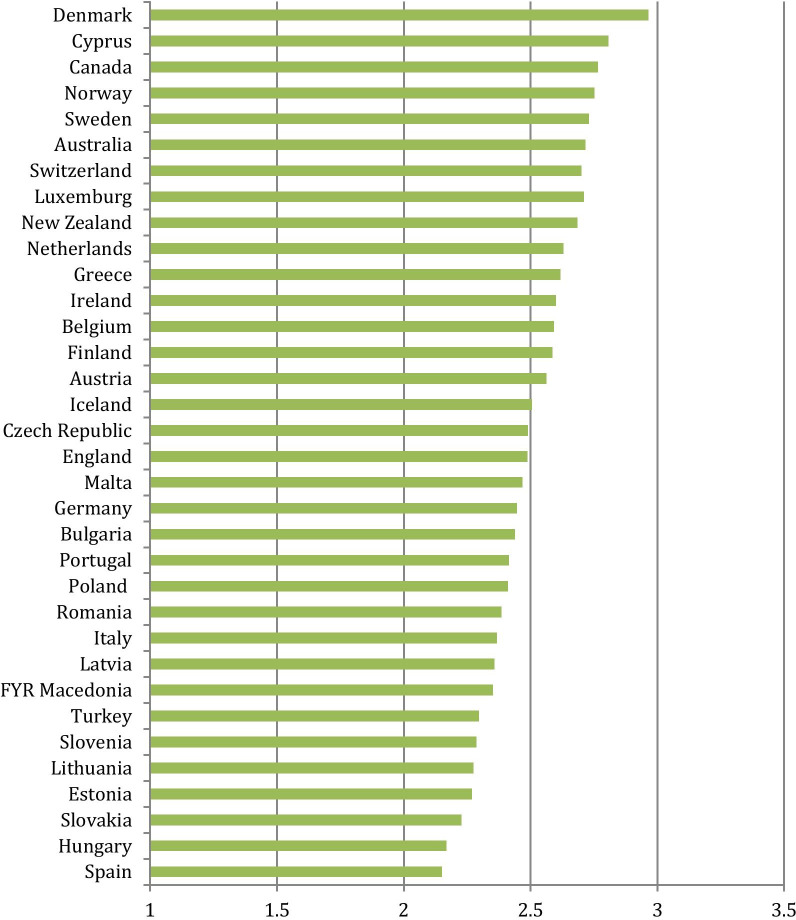
Mean level of job satisfaction per country (on a scale of 1–4)

Cronbach’s alphas for the job satisfaction items were 0.62 at the GP level and 0.97 at the country level. At GP level, the reliability of the scale is fair; at country level the reliability is very strong (related to the high ICC). The job satisfaction scale is normally distributed (mean and median are the same (2,5), skewness is -0,02 and kurtosis is 2,7 (which is very close to the normal distribution with skewness = 0 and kurtosis = 3); 90% of the observations lies between 1,9 and 3,1). The countries in this study substantially differ with regard to GP job satisfaction. Denmark, Cyprus, Canada and Norway have the highest mean scores, while Spain, Hungary, Slovakia and Estonia have the lowest mean scores (Fig. 1).

Table 2 shows that the variation between countries in the empty model is 33% of the total variation. The fixed part of Table 2 (the regression coefficients of the independent variables) shows that GPs who carry out more technical procedures and who are more involved in preventive care and health promotion are more satisfied with their job. GPs who work more hours are less satisfied, while GPs who take more weeks of vacation per year are more satisfied. GPs who receive more feedback from their colleagues have higher job satisfaction. GPs with more satisfied patients are more satisfied with their job as well. The interaction between patient satisfaction and shared practice was not significant (not in table). Finally, older GPs are more satisfied. Overall, when Model 5 is compared to Model 0, the analysis shows that the full model explains only just over 1% of the variance on the GP and practice level. The reduction in variance at country level between the full model and the empty model is around 13%. This is due to the clustering of GP and practice characteristics within countries. Examination of in-between models (not in table) suggests that this is mainly caused by the fact that performance of technical procedures is related to job satisfaction and different between countries.

**Table 2:** Multilevel linear regression results of the interrelation between GP and practice characteristics and GP job satisfaction (N_country_ = 34; N_GPs_ = 7,379)

	M0: B (S.E.)	M5: B (S.E.)
Fixed coefficients		
Constant	2.505 (.034)***	2.370 (.059)***
Stimulation		
Breadth of service profile: First contact care Management of disease Technical procedures Health promotion		.009 (.009) .000 (.009) .028 (.008)*** .061 (.024)**
Medical equipment		.001 (.001)
Other paid activities (ref = no other paid activities)		.003 (.008)
Practice location (ref = big inner city) Suburbs or small town Urban–rural or rural		− .000 (.009) − .005 (.009)
Comfort		
Working hours		− .001 (.000)***
Administrative work		− .000 (.000)
Hours spent in out-of-hours work		.000 (.000)
ICT use		.000 (.002)
Vacation		.007 (.002)**
Practice building (ref = not clean and no privacy) Clean or private Clean and private		.000 (.022) .001 (.022)
Status		
Self-employed (ref = salaried)		.000 (.011)
Behavioural confirmation		
Feedback from colleagues (ref = no feedback)		.027 (.008)**
Patient satisfaction		.095 (.032)**
Shared practice (ref = solo)		.001 (.008)
Control variables		
Age		.000 (.000)**
Gender (ref = male)		− .013 (.007)
Random coefficients		
Level: country variance	.038 (.009)***	.033 (.008)***
Level: GP/practice variance	.078 (.001)***	.077 (.001)***
N: country	34	34
N: practice	7379	7379
ICC	.329	.302
− 2*log-likelihood	2294.18	2139.39
Change in − 2LL (df)		− 154.79 (28)***

***p < .001; **p < .05

As shown in Table 3, of all the country variables that were tested, only GDP per capita is strongly related to GP job satisfaction. When Model 6 is compared to Model 5, the decrease in country-level variance shows that 33% of the remaining country-level variance is explained. Since GDP per capita is the only variable that was added as compared to Model 5, this shows that GDP per capita explains one-third of the country-level variance. None of the health system-related variables at country level that were tested are significantly related to GP job satisfaction.

**Table 3:** Multilevel linear regression results of the interrelation between country characteristics and GP job satisfaction (all GP- and practice-level variables included)

	M6: B (S.E.)	M7: B (S.E.)	M8: B (S.E.)	M9: B (S.E.)	M10: B (S.E.)
y = job satisfaction					
Fixed coefficients					
Constant	2.212 (.067)***	2.209 (.395)***	2.210 (.092)***	2.213 (.069)***	2.307 (.093)***
Level: country					
GDP per capita	.000 (.000)***	.000 (.000)***	.000 (.000)***	.000 (.000)***	.000 (.000)**
Strength of primary care		.001 (.173)			
Out-of-hours model (ref = small family doctor based)					
Large family doctor based			-.007 (.075)		
Hospital- and national based			.002 (.068)		
Relative income position (ref = low)					
Medium				.001 (.081)	
High				− .009 (.068)	
Patient list system (ref = no patient list system)					− .091 (.062)
Random coefficients					
Level: country variance	.022***	.022***	.022***	.022***	.020***
Level: practice variance	.077***	.077***	.077***	.077***	.077***
N: country	34	34	34	34	34
N: practice	7379	7379	7379	7379	7379
ICC	.221	.221	.221	.221	.211
− 2*log-likelihood	2125.25	2125.25	2125.23	2125.23	2123.18
Change in -2LL (df)	M5: − 14.14 (1)***	M6: − 0.00 (1)	M6: − 0.02 (2)	M6: − 0.02 (2)	M6: − 2.07 (1)***

***p < .001; **p < .05; *p <  .10

## Discussion

Job satisfaction of GPs differs between countries. One-third of the variation in GP job satisfaction is between countries. GPs in the Scandinavian countries and the Netherlands are in the upper-third of average job satisfaction, while GPs in Southern European countries are in the lower-third. The average values for GP job satisfaction per country have no absolute meaning, but derive their meaning from the comparison.

We developed hypotheses to explain variation in job satisfaction based on SPF theory. We theorised that GPs ‘produce’ job-related well-being through doing stimulating work, deriving comfort, social status and behavioural confirmation from their work. Some of our hypotheses related to stimulation were confirmed in the analysis. GPs who perform more technical procedures or are more involved in preventive services and health promotion are more satisfied with their job. Concerning comfort, as hypothesised the number of working hours per week is negatively related to GP job satisfaction and the number of weeks of vacation per year positively. None of the hypotheses relating to status as means to produce job satisfaction was confirmed. However, hypotheses concerning behavioural confirmation were confirmed. The analysis shows that receiving feedback from colleague GPs and higher patient satisfaction are related to higher GP job satisfaction. Finally, the control variable age was found to be positively related to job satisfaction; older GPs are more satisfied. This may be partly explained by the ‘healthy worker effect’ [66]; older GPs who are satisfied may tend to stay in work longer, while less satisfied GPs retire earlier and are therefore not included in this study. The wealth of countries, which we included as a potential confounder, turned out to be the only variable at country level that is related to GP job satisfaction. The health system variables at country level, related to our hypotheses, were not related to job satisfaction of GPs. The rank order of countries by GP job satisfaction may reflect job satisfaction in the working population in general. This seems to be more so at the bottom end of the ranking than at the top of the ranking, when comparing with survey data on job satisfaction among employees in general [67]. The relationship between a country’s wealth and satisfaction is a general effect, known from the literature [68]. However, behind the association between GDP per capita and life satisfaction other cultural and institutional mechanisms may hide, such as individualism, equality and human rights [69].

Some relationships that we have hypothesised have not been found in this study. With regard to *stimulation*, the hypothesis about the range of services provided was only partly confirmed. The hypothesis about equipment, a resource to produce a broad range of services, was refuted. We also did not find the hypothesised relationship for paid activities apart from the GP practice. We assumed that these activities would increase task variety, skill improvement and positive feedback from students and would in turn increase job satisfaction through more stimulation and behavioural confirmation. However, it could be that that some GPs mainly perform these tasks for the additional income, while they do not really enjoy these tasks. Practice location was also unrelated to job satisfaction. GPs in rural areas have a broader range of tasks [24], but this may be overridden by a higher workload [70]. Another secondary analysis of the QUALICOPC data showed a broader range of tasks, and higher workload for rural GPs, but no difference in job satisfaction between rural and urban GPs [71]. In the area of *comfort*, a surprising finding is that there is no significant relationship between administrative work and GP job satisfaction, especially since this was found to be one of the most important factors that decreases job satisfaction [72]. Due to limitations in the data, administrative work was measured by subtracting the amount of hours spent on direct patient care from the total amount of working hours, with the assumption that most of this time would be spent on administrative work. The hours remaining after direct patient care is an heterogeneous category, also containing satisfaction enhancing activities, such as keeping up to date [73]. Another possibility is that administrative and organisational tasks increasingly become an accepted part of professionals’ work [74]. GPs might not enjoy these tasks very much, but not resist them either because they acknowledge that it is an important aspect of their work. Our hypothesis about out-of-hours work, which is often mentioned as a source of work stress and dissatisfaction, was not confirmed; neither at the GP level in terms of hours spent nor at health system level in the form of a classification of the organisation of out-of-hours care. The hypotheses about the use of information technology, as a resource to make work easier, and about the practice premises were not confirmed. We tested two hypotheses related to the *status* of GPs and neither was confirmed. Self-employed GPs are not more satisfied than salaried GPs and GPs in countries with a good income position relative to other physicians are not more satisfied. The absence of a relationship with employment status could perhaps be clarified by taking into account whether GPs prefer to be self-employed or salaried. A Norwegian study has found that while a majority of GPs are and prefer to be self-employed there is also a significant minority that would prefer a salaried position [75]. With regard to *behavioural confirmation*, we found that GP job satisfaction is not related to the type of practice (solo or shared).This suggests that the positive effect of behavioural confirmation does not differ between GPs from solo- or shared practices. It also suggests that feedback does not have to be given by direct colleagues who work in the same practice, but that it can also be provided by GPs from other practices. Overall and in terms of our theoretical approach, we found that GPs are able to ‘produce’ work-related well-being through activities and resources related to stimulation, comfort and behavioural confirmation, but not to status.

We should note that the explanatory power in the sense of explaining variation of our regression models is small at the GP and practice level. In contrast, GDP per capita explains one-third of the variation between countries. Although this shows a large explanatory power at country level in the statistical sense, we were not able to explain country variation in the theoretical sense of confirmed hypotheses about health system characteristics. All the other country-level variables that were tested did not show a significant relationship with GP job satisfaction. It is possible that the health system variables that we used, do not capture important influences on GP job satisfaction and that other health system features would have done.

Our study has a number of strengths. The first strength is the theoretical approach to GP job satisfaction. The SPF theory is an attractive framework, because it emphasises agency in the form of goal-oriented behaviour. Many of the variables in the hypotheses we tested refer to modifiable variables, either by GPs themselves or at a collective level by professional organisations and policymakers [76]. This means that targeted interventions could result in higher job satisfaction. A further advantage of our approach is that other variables that can be subsumed under the theoretical categories, such as behavioural confirmation where we found, e.g. associations with peer feedback, might also increase job satisfaction. A second strength is the use of a large international data set, although the fact that this study was not designed to study job satisfaction at the same time is a weakness. We used a state-of-the-art statistical approach by performing random effects multilevel analysis, which takes the nested structure of the data into account [65].

There are also weaknesses. It should be noted that cross-sectional nature of the data only show how variables are related to each other, and cannot indicate causal relationships. This means that it is not possible to say whether statistically significant variables would cause a change in GP job satisfaction. This also means that certain variables, such as patient satisfaction or performing certain services, could be dependent on GP job satisfaction instead of the other way around, or that they influence each other. Secondly, it should be noted that this study could be subject to sample selection bias, as it could be that GPs who are more satisfied are also more willing to participate in a large survey. However, this does not necessarily have to be the case since job satisfaction was not the main aim of the survey and it was only mentioned in one out of the 60 questions. The questionnaire of the QUALICOPC study did not include psychological variables, such as coping abilities; hence, there is risk of misspecification in the sense that some of the relations we found, e.g. the relationship with working hours, could actually relate to the psychological abilities of GPs to cope with their workload.

When designing new, international comparative research specifically into job satisfaction of GPs, several points come up from this secondary analysis. First of all, the measurement of job satisfaction could be improved. The six items to measure job satisfaction that were used in the QUALICOPC study, were taken from a previous study. However, in a new and specific study on GP job satisfaction, a comparison should be made with other existing instruments. Moreover, the measurement of job satisfaction could linked to the SPF theory as has been done for general well-being [78]. A survey among GPs on job satisfaction should also include more attention to psychological determinants of job satisfaction (as mentioned in the previous paragraph). Finally, it would be important both from a scientific and a policy standpoint, to have a wider exploration of the international differences in job satisfaction. This could be done by also studying another population than GPs to find out to what extent international differences in job satisfaction are specific for GPs or more general for large groups of the working population.

In conclusion, a high level of job satisfaction of GPs is important because it may help to maintain the current workforce and to make general practice a more appealing career option for new doctors [77]. The finding that a broad range of services is related to higher job satisfaction suggests that GP job satisfaction could be increased by stimulating GPs to spend more time on, e.g. technical procedures and health promotion. The negative relationship between work hours and job satisfaction is important in view of the work–life balance which has become increasingly important for GPs, especially for younger and female GPs [79], which suggests that this issue will be even more important in the future. Feedback from colleagues and patients could be strengthened by policies at practice level as well as regional or national level. However, the evidence base for policy development is still rather small. Finally, the strong relationship between GDP per capita and GP job satisfaction should be examined further. Given the clustering of GP job satisfaction within countries, national interventions aimed at increasing job satisfaction can be very useful, but these can only be developed if we have more insight in (health)policy-amenable influences. Therefore, further research into differences in job satisfaction between countries is recommended.

## Supplementary Information

**Additional file 1**: **Table S1.** Independent variables: GP- and practice level. **Table S2.** Independent variables: country level. **Table S3.** Table S3: Descriptive information on the independent variables.

## Data Availability

The datasets used and/or analysed during the current study are available from the corresponding author on reasonable request.

## Abbreviations

FYR Macedonia: Former Yugoslav Republic of Macedonia
GDP: Gross Domestic Product
GP(s): General practitioner(s)
ICC: Intraclass correlation
QUALICOPC: Quality and Costs of Primary Care in Europe
SPF Theory: Social Production Function Theory
