# Supplementary material for: Job satisfaction of general practitioners: a cross-sectional survey in 34 countries
Source: Hum Resour Health. 2021 Apr 27;19:57. doi: 10.1186/s12960-021-00604-0 (PMC8077953; doi:10.1186/s12960-021-00604-0)
Supplement: Supplementary file 1 — Additional file 1: Table S1. Independent variables: GP- and practice level. Table S2. Independent variables: country level. Table S3. Table S3: Descriptive information on the independent variables. [file 12960_2021_604_MOESM1_ESM.docx]

**Supplementary tables**

**Supplementary table 1: Independent variables: GP- and practice level**

| **Variable** | **Operationalisation** | **Measurement** | **Missing values** |
| --- | --- | --- | --- |
| Range of services  1. First contact care, scale  2. Treatment of (mainly chronic) diseases, scale  3. Technical procedures, scale  4. Preventive services and health promotion, scale |  |  |  |
|  | To what extent will patients in your practice population contact you as the first health care provider? 19 items | Recoded into a continuous variable with range 1-4 for which 1 = low first contact care; 4 = high first contact care | 0.63%, 47 cases missing (distributed over 17 countries, most notably Slovakia with 10 missing cases) |
|  | To what extent are you involved in the treatment and follow-up of patients in your practice population with the following diagnoses? 12 items | Recoded into a continuous variable with range 1-4 for which 1 = low treatment and follow-up; 4 = high treatment and follow-up | 0.73%, 54 cases missing (distributed over 20 countries, most notably Slovakia with 15 missing cases) |
|  | To what extent are the following activities carried out in your practice population by you, or your staff, and not by a medical specialist? 10 items | Recoded into a continuous variable with range 1-4 for which 1 = few technical procedures; 4 = many technical procedures | 0.63%, 47 cases missing (distributed over 19 countries, most notably Slovakia with 12 missing cases) |
|  | **-** When do you, or your staff, measure blood pressure?  **-** When do you, or your staff, measure blood pressure? **-** To what extent are you involved in health education as regards the following topics? [Smoking / Diet / Problematic use of alcohol] | Recoded into a continuous variable with range 1-4 for which 1 = low prevention; 4 = high prevention | 0.39%, 29 cases missing (distributed over 17 countries, most notably Slovakia and Finland with both 5 missing cases) |
| Medical instruments, sum score | Please tick the equipment used in your practice by yourself or your staff [...], 30 items | Recoded into a continuous variable with range 0-30 in absolute number of instruments | 0.49%, 36 cases missing (distributed over 12 countries, most notably Bulgaria with 12 missing cases) |
| Other paid professional activities | Beside your work as a GP in this practice, do you have any other paid professional activities? [No; yes, as a private physician/in residential setting/company doctor/medical education/other] | Recoded into a dummy variable with reference category = no paid side activities; dummy1 = paid side activities; dummy2 = missing | 13.7%, 1,019 cases missing (distributed over 33 countries, most notably Canada with 113 missing values) |
| Practice location | How would you characterise the place where you are currently practising? [big (inner)city / suburbs / (small) town / mixed urban-rural / rural] | Recoded into 3 dummy variables with reference category = big (inner) city;dummy1 = suburbs or small town; mixed urban-rural or rural; dummy2 = missing | 1.1%, 84 cases missing (relatively evenly distributed over 26 countries) |
| Working hours | How many hours per week do you work as a GP (excluding additional jobs and on-call or out-of-hours services)? | Recoded into a variable with range 1-75 in absolute hours per week, relative to the mean number of working hours within a country | 1.96%, 145 cases missing (distributed over 27 countries, most notably in Belgium and Canada with 28 and 23 cases missing) |
| Administrative work | How many of these hours do you spend on direct patient care (consultations, home visits, telephone consultations)? | No direct measurement of hours spent on administrative work. Proxy measurement by the proportion of hours GPs don’t spend on direct patient care | 1.96%, 145 cases missing (distributed over 27 countries, most notably Canada and Belgium with 61 and 35 cases missing) |
| Out-of-hours work | In the past 3 working months (excluding holidays, etc.), how often and for how long did you have on-call duties during evenings, nights) and weekends?  **-** During evening(s) _ times; in total _ hours;  **-** During night(s)_ times; in total _ hours  **-** During weekend days _ times; in total _ hours | Recoded into a ratio variable with range 0 - 1668 in absolute hours of total out-of-hours work | 15.92%, 1,180 cases missing (distributed over 34 countries, most notably Canada and Latvia with 147 and 122 cases missing)  (The missing values on the out-of-hours variable were recoded to 0 in order to maintain cases. It is assumed that GPs who did not answer this question did not perform any out-of-hours work) |
| ICT use | For which of the following purposes do you use a computer in your practice? [Not applicable; making appointments; issuing invoices; issuing drug prescriptions; keeping records of consultations; sending referral letters to medical specialists; storing diagnostic test results; searching medical information on the internet; sending prescriptions to the pharmacy] | Recoded into a ratio variable with range 0 - 8 in number of purposes of computer use | 2.21%, 164 missing cases (distributed over 19 countries, most notably Lithuania and New Zealand and with 73 and 61 cases missing) |
| Vacation | In the past 12 months, about how many weeks altogether have you been away from the practice due to vacation? | Recoded into a ratio variable with range 0 – 35, relative to the mean number of weeks within a country | 2.09%, 155 missing cases (distributed over 27 countries, most notably Slovakia and Latvia with 29 and 24 cases missing) |
| Practice building | **-** How clean does the waiting room look? [Very clean; rather clean; not clean]  Recoded into a dummy variable with 0 = not or rather clean; 1 = very clean.  **-** Can people in the waiting room hear or see what happens in the doctor’s office? [Yes; no]  Dummy variable with 0 = no privacy; 1 = privacy | Recoded into dummy variables with reference category = not clean and no privacy; dummy1 = clean or private; dummy2 = clean and private; dummy3 = missing | 12.19%, 904 missing cases (distributed over 32 countries, most notably Denmark, New Zealand, Finland and Australia with respectively 212, 168, 153 and 152 cases missing) |
| Employment status | As a GP, are you self-employed or in salaried employment? [Salaried employment with centre or authority; salaried employment with other GP; Self-employed with contract(s) with health service, insurance or authority; Self-employed without contract(s)] | Recoded into a dummy with 0 = salaried employment with centre or authority; salaried employment with other GP; and 1 = self-employed with contract(s) with health service, insurance or authority; self-employed without contract(s) | 1.42%, 105 cases missing (distributed over 26 countries, most notably Lithuania and Spain with 14 and 13 missing cases) |
| Shared or solo practice | Do you work alone or in shared accommodation with one or more GPs and/or medical specialists? [alone; with _ other GPs in shared accommodation; with _ other medical specialist(s) in shared accommodation | Recoded into a dummy variable with reference category = solo practice; dummy1 = shared practice; dummy2 =  missing | 0.97%, 72 cases missing (distributed over 17 countries, most notably Belgium, Finland and Latvia with 18, 16 and 13 missing cases) |
| Feedback from colleague GPs | In the past 12 months, has the following occurred in your practice/centre: Feedback from colleague GPs (peer review or practice visitation)? [yes / no] | Recoded into a dummy variable with 0 = no feedback; 1 = feedback | 1.69%, 125 missing cases (distributed over 27 countries, most notably Bulgaria, Spain and Latvia with 13, 13 and 12 missing cases) |
| Patient satisfaction | I would recommend this doctor to a friend or relative [yes / no] | Proportion of patients per GP that would recommend their doctor | 4.33%, 321 missing cases (relatively evenly distributed over 34) |
| GP age (potential confounder) | What is your year of birth? | Continuous variable | 1.0%, 69 missing cases (relatively evenly distributed over 26 countries) |
| GP gender (potential confounder) | Are you male or female? | Dummy variable, reference category = male; 1= female | 0.41%, 28 missing cases (relatively evenly distributed over 15 countries) |

**Supplementary table 2: Independent variables: country level**

| **Variable** | **Operationalisation** | **Missing values** | **Data source(s)** |
| --- | --- | --- | --- |
| GDP per capita (potential confounder) | US dollars ($) | 0 cases missing | World Bank (2016) |
| Strength of primary care system | Strength of primary care on a scale 1 - 3 for which 1 = low primary care orientation and 3 = high primary care orientation | 0 cases missing | Kringos (2013) |
| Out-of-hours care | Categorical variable with 1 = small family doctor based model; 2 = large family doctor based model; 3 = hospital- and national based model | 0 cases missing | Huibers et al. (2009); Dimova et al. (2012); Theodorou et al. (2012); Lai et al. (2013); Vuorenkoski et al. (2008); Gaál et al. (2011); Mitenbergs et al. (2012); Murauskiene et al. (2013); Berthet et al. (2015); Milevska-Kostova et al. (2017); Azzopardi-Muscat et al. (2017); Sagan et al. (2011); Vlădescu et al. (2016); Smatana et al. (2016); WHO (2012) |
| Relative income position | GP income level compared to most specialists’ income in three categories with 1 = low; 2 = medium; 3 = high | 0 cases missing | Kringos et al. (2015: chapter 2) |
| Patient list system | Dummy with 1 = patient list system; 0 = no patient list system | 0 cases missing | Kringos et al. (2015: chapter 3); Schäfer 2016 |

**References to Supplementary table 2**:

Azzopardi-Muscat N, Buttigieg S, Calleja N, Merkur S (2017). Malta: Health system review. Health Systems in Transition, 2017; 19(1):1–137.

Berthet F, Calteux A, Wolter M et al. Luxembourg: Health systems in transition; HiT in brief. Eurpepean Observatory on Health Systems and Policies, 2015.

Dimova A, Rohova M, Moutafova E, Atanasova E, Koeva S, Panteli D, van Ginneken E. Bulgaria: Health system review. Health Systems in Transition, 2012, 14(3):1–186.

Gaál P, Szigeti S, Csere M, Gaskins M, Panteli D. Hungary: Health system review. Health Systems in Transition, 2011; 13(5):1–266.

Huibers L, Giesen P, Wensing M, Grol R. Out-of-hours care in western countries: assessment of different organizational models. BMC health services research 2009;9: 105.

Kringos DS, Boerma WGW, Van der Zee J, Groenewegen PP. Political, cultural and economic foundations of primary care in Europe. Social Science & Medicine 2013, 99:9-17.

Kringos DS, Boerma WGW, Hutchinson A, Saltman RB. Building primary care in a changing Europe. WHO Regional Office for Europe, Copenhagen, 2015.

Lai T, Habicht T, Kahur K, Reinap M, Kiivet R, van Ginneken E. Estonia: health system review. Health Systems in Transition, 2013; 15(6):1–196.

Milevska Kostova N, Chichevalieva S, Ponce NA, van Ginneken E, Winkelmann J. The former Yugoslav Republic of Macedonia: Health system review. Health Systems in Transition, 2017; 19(3):1–160.

Mitenbergs U, Taube M, Misins J, Mikitis E, Martinsons A, Rurane A, Quentin W. Latvia: Health system review. Health Systems in Transition, 2012; 14(8): 1 – 191.

Murauskiene L, Janoniene R, Veniute M, van Ginneken E, Karanikolos M. Lithuania: health system review. Health Systems in Transition, 2013; 15(2): 1–150.

Sagan A, Panteli D, Borkowski W et al. Poland: Health system review. Health Systems in Transition, 2011, 13(8):1–193.

Schäfer WLA. Primary care in 34 countries: perspectives of general practitioners and their patients. PhD thesis Utrecht University; Utrecht, Nivel, 2016. Available from: <https://dspace.library.uu.nl/bitstream/handle/1874/341143/Sch_fer.pdf?sequence=1&isAllowed=y>

Smatana M, Pažitný P, Kandilaki D, Laktišová M, Sedláková D, Palušková M, van Ginneken E, Spranger A (2016). Slovakia: Health system review. Health Systems in Transition, 2016; 18(6):1–210.

Theodorou M, Charalambous C, Petrou C, Cylus J. Cyprus: Health system review. Health Systems in Transition. 2012; 14(6):1–128.

Vlãdescu C, Scîntee SG, Olsavszky V, Hernández-Quevedo C, Sagan A. Romania: Health system review. Health Systems in Transition, 2016; 18(4):1–170.

Vuorenkoski L, Mladovsky P and Mossialos E. Finland: Health system review. Health Systems in Transition. 2008; 10(4): 1–168.

World Bank. GDP per capita (current US$) 2016. Retrieved April 30, 2018, from

<https://data.worldbank.org/indicator/NY.GDP.PCAP.CD>

WHO. Turkey Health System Performance Assessment 2011. WHO/World Bank 2012.

Supplementary table 3: Descriptive information on the independent variables (N_country_=34; N_GPs_=7,379).

|  | Percentage | Mean (standard deviation) | Median (interquartile range) | Number of missing observations |
| --- | --- | --- | --- | --- |
| ***GP/Practice characteristics*** |  |  |  |  |
| Breadth of service profile:  First contact care  Management of disease  Technical procedures  Health promotion |  | 2.9 (0.5)  3.3 (0.49)  2.14 (0.79)  0.19 (0.16) | 3.0 (2.6-3.32)  3.4 (2.96-3.72)  2.0 (1.42-2.77)  0.17 (0.07-0.27) | 47  54  47  29 |
| Medical equipment |  | 14.7 (5) | 14 (11-18) | 36 |
| Other paid activities – percentage Yes | 35% |  |  | 1,019 |
| Practice location   - big (inner) city - Suburbs or small town - Mixed urban-rural or rural | 31%  35%  34% |  |  | 84 |
| Working hours |  | 100 (24) | 100 (89-112) | 145 |
| Administrative work |  | 19 (21) | 14 (0-25) | 145 |
| Hours spent in out-of-hours work |  | 60 (139) | 5 (0-56) | 1,180 |
| ICT use |  | 5.2 (2.3) | 6 (4-7) | 164 |
| Vacation |  | 3.8 (1.8) | 4 (2-5) | 155 |
| Practice building   - not clean and no privacy - Clean or private - Clean and private | 3%  36%  61% |  |  | 904 |
| Self-employed – percentage Yes | 65% |  |  | 105 |
| Feedback from colleagues – percentage Yes | 30% |  |  | 125 |
| Patient satisfaction |  | 0.94 (0.11) | 1 (0.89-1) | 321 |
| Shared practice – percentage Yes | 60% |  |  | 72 |
| Age of GP |  | 50 (9.7) | 51 (43-58) | 69 |
| Gender – percentage Female | 52% |  |  | 28 |
| ***Country characteristics*** |  |  |  |  |
| GDP per capita |  | 33147 (19557) | 30669 (16530-43433) | 0 |
| Strength of primary care |  | 2.27 (0.13) | 2.28 (2.17-2.36) | 0 |
| Out-of-hours model  - Small family doctor based  - Large family doctor based  - Hospital- and national based | 24%  18%  57% |  |  | 0 |
| Relative income position  - Low  - Medium  - High | 73%  10%  17% |  |  | 0 |
| Patient list system – percentage Yes | 67% |  |  | 0 |
